# Histamine poisoning and control measures in fish and fishery products

**DOI:** 10.3389/fmicb.2014.00500

**Published:** 2014-09-23

**Authors:** Pierina Visciano, Maria Schirone, Rosanna Tofalo, Giovanna Suzzi

**Affiliations:** Faculty of BioScience and Technology for Food, Agriculture and Environment, University of TeramoMosciano Sant’Angelo, Italy

**Keywords:** histamine, fishery products, food poisoning, regulation

## Abstract

Histamine poisoning is one of the most common form of intoxication caused by the ingestion of fish and fishery products. Cooking, canning, or freezing cannot reduce the levels of histamine because this compound is heat stable. All humans are susceptible to histamine and its effects can be described as intolerance or intoxication depending on the severity of the symptoms. The amount of histamine in food, the individual sensitivity, and the detoxification activity in human organism represent the main factors affecting the toxicological response in consumers. Histamine is the only biogenic amine with regulatory limits set by European Legislation, up to a maximum of 200 mg/kg in fresh fish and 400 mg/kg in fishery products treated by enzyme maturation in brine.

## INTRODUCTION

Histamine is a biogenic amine produced in fish tissue through the decarboxylation of free histidine by exogenous decarboxylases released by microorganisms. This ability has been described in different genera, species, and strains of bacteria, both Gram positive and Gram negative (Ladero et al., 2010). Histamine is rarely found in fresh fish but its level increases with the progress of fish decomposition (Shakila et al., 2003). The microorganisms naturally present on the gills and in the gut of live fish start to grow upon death because the defense mechanisms are inactive. In particular histamine forming bacteria are able to grow more rapidly at high abuse than at moderate abuse temperatures. However once the enzyme histidine decarboxylase has been formed, it can continue to produce histamine also at or near refrigeration temperature, it remains stable in frozen fish and can be reactivated after thawing. Frozen temperature (-18°C or below) can stop the growth of bacteria and prevent any preformed histidine decarboxylase from producing histamine. Conversely histamine production is greater at high abusive temperatures (21.1°C or higher) particularly at temperatures near 32.2°C [Food and Drug Administration (FDA), 2011]. Cooking can inactivate both the enzyme and the microorganisms, but histamine which has been formed cannot be eliminated because it is heat stable [Food and Drug Administration (FDA), 2011].

Histamine poisoning is a food-borne disease characterized by a variety of symptoms similar to allergic reactions. The toxic effects of histamine are related to its normal physiological actions in the body. In particular the dilatation of the peripheral blood vessels results in hypotension, flushing, and headache, while the increased capillary permeability causes urticaria, hemoconcentration, and eyelids edema; the symptoms affecting the gastrointestinal system are due to the contraction of smooth muscles leading to abdominal cramps, diarrhea, nausea, and vomiting. Histamine exerts also a stimulatory action on the heart by increasing its contractility and exhibiting palpitations and tachycardia, while it is a potent stimulant of sensory and motor neurons producing pain and itching associated with the rash [Food and Agriculture Organization/World Health Organization (FAO/WHO), 2012].

The variability of symptoms can be linked both to the amount of histamine ingested and individual sensitivity. The ingestion of food containing small amounts of histamine has little effect in healthy individuals, but it can result in histamine intolerance in persons characterized by impairment of diamine oxidase (DAO) activity, either due to genetic predisposition, gastrointestinal diseases, or medication with monoamine oxidase (MAO) inhibitors (Maintz and Novak, 2007), whereas histamine intoxication can appear in everyone as a result of its high amounts in foods like fish and fishery products or ripened cheese [European Food Safety Authority (EFSA), 2011]. The symptoms of histamine poisoning can appear for few hours or a day but in rare case they can persist for some days. However, statistical data about its incidence are not available because the poisoning incidents are often underestimated due to mild or not recognized nature of illness and to inadequate systems to attribute food-borne diagnosis.

The histamine intoxication outbreaks between 2005 and 2010 using Rapid Alert System for Food and Feed (RASFF) were above 100 cases [European Food Safety Authority (EFSA), 2011]. The measures used to estimate the dose/exposure level causing histamine intoxication are generally based on the detection of the biogenic amine in the suspected food or on the patients reports. Moreover, the toxic effects of histamine are enhanced by the presence of other biogenic amines such as putrescine and cadaverine (Huang et al., 2010).

## REGULATORY LIMITS

According to Commission Regulation EC No 2073/2005 (Regulation 2073/2005/EC) the limits for histamine have been established in fish species associated with a high amount of histidine, i.e., the families of *Scombridae*, *Scombresosidae*, *Clupeidae*, *Engraulidae*, *Coryfenidae*, *Pomatomidae*, both fresh and treated by enzyme maturation in brine. The sampling plan consists of a number of units comprising the sample (*n*) equal to nine and a number of sample units (*c*) equal to two, giving values between *m* and *M*. In particular the examined batch will be satisfactory when: (i) the mean value is less or equal to *m*; (ii) a maximum of *c*/*n* values are between *m* and *M*; (iii) no values exceed *M*. The sampling scheme of Food and Drug Administration offers more confidence that non-conforming lots will be detected, as reported in **Table 1**. Moreover EU assessed the limits of histamine for fishery products which have undergone enzyme maturation with brine manufactured from fish species associated with a high amount of histidine equal to 200 mg/kg and 400 mg/kg, for *m* and *M*, respectively.

**Table 1:** Sampling schemes for histamine content in fishery products.

Sampling plan	*n*	*c*	Histamine (mg/kg)	
			*m*	*M*
European Union*	9	2	100	200
Food and Drug Administration	18	1	50	500

*Scombridae, Scombresosidae, Clupeidae, Engraulidae, Coryfenidae, Pomatomidae.

For the detection of histamine also single samples may be taken at retail level. In such cases the whole batch should not be deemed unsafe based only on the result of one sample, unless the result is above *M*, as reported in Commission Regulation EU No 1019/2013 (Regulation 1019/2013/EU). The last Regulation amended Annex I to Regulation EC No 2073/2005 adding a maximum value for fish sauce produced by fermentation of fishery products, equal to 400 mg/kg. Since fish sauce is a liquid fishery product, histamine can be expected to be evenly distributed then a single sample can be examined.

The reports on histamine intoxication generally involve only a small number of individuals, so it is difficult to estimate the dose/exposure level in order to construct quantitative assessment of dose versus adverse response. A model used in the dose/response assessment can be based on volunteer challenge study, as reported by the EFSA biogenic amines report [European Food Safety Authority (EFSA), 2011]. In particular these studies aim to investigate the minimal dose of histamine that causes poisoning or intolerance symptoms, carefully monitored by medical professionals. Results from the limited number of studies suggested a potential no observed adverse effect level (NOAEL) of 50 mg histamine for the symptoms headache and flushing, but this was based on limited number of individuals: 66 healthy and 74 sensitive. Some healthy individuals did not show symptoms at concentrations up to six times higher than the NOAEL [European Food Safety Authority (EFSA), 2011]. Based on the above mentioned NOAEL and the consumption of a portion/size of 250 g of fish, the maximum concentration of histamine that would not cause an adverse effect would be equal to 200 mg/kg [Food and Agriculture Organization/World Health Organization (FAO/WHO), 2012].

## PREVENTIVE AND HYGIENIC MEASURES

The risk of histamine poisoning could be controlled by applying basic Good Manufacturing and Hygiene Practices associated to an appropriate Hazard Analysis Critical Control Point (HACCP) system. According to European Legislation fish must be maintained at a temperature approaching that of melting ice as soon possible after harvest, in order to comply with freshness criteria and to avoid the growth of spoilage and histamine producing bacteria. All operations (heading, gutting, filleting, or cutting, etc.) should be carried out hygienically on board vessels. Moreover fresh fishery products must be kept at the above mentioned temperature during storage and transport in such a way as not adversely to affect food safety.

## CONCLUSION

Fishery products can be involved in foodborne outbreaks by histamine when the application of Good Hygiene Practices and proper temperatures of storage failed to comply during the food chain. The regulatory systems have developed control strategies and monitoring procedures, such as sampling plan for fish species with a high amount of histidine, in order to assuring seafood safety.

## Conflict of Interest Statement

The authors declare that the research was conducted in the absence of any commercial or financial relationships that could be construed as a potential conflict of interest.
